# Dysregulation of the Intrarenal Vitamin D Endocytic Pathway in a Nephropathy-Prone Mouse Model of Type 1 Diabetes

**DOI:** 10.1155/2011/269378

**Published:** 2011-05-16

**Authors:** John L. Fowlkes, R. Clay Bunn, Gael E. Cockrell, Lindsey M. Clark, Elizabeth C. Wahl, Charles K. Lumpkin, Kathryn M. Thrailkill

**Affiliations:** Arkansas Children's Hospital Research Institute, Department of Pediatrics, University of Arkansas for Medical Sciences, 1 Children's Way, Slot 512-6, Little Rock, AR 72202, USA

## Abstract

Microalbuminuria in humans with Type 1 diabetes (T1D) is associated with increased urinary excretion of megalin, as well as many megalin ligands, including vitamin-D-binding protein (VDBP). We examined the DBA/2J diabetic mouse, nephropathy prone model, to determine if megalin and VDBP excretion coincide with the development of diabetic nephropathy. Megalin, VDBP, and 25-hydroxy-vitamin D (25-OHD) were measured in urine, and genes involved in vitamin D metabolism were assessed in renal tissues from diabetic and control mice at 10, 15, and 18 weeks following the onset of diabetes. Megalin, VDBP, and 25-OHD were increased in the urine of diabetic mice. 1-*α* hydroxylase (CYP27B1) mRNA in the kidney was persistently increased in diabetic mice, as were several vitamin D-target genes. These studies show that intrarenal vitamin D handling is altered in the diabetic kidney, and they suggest that in T1D, urinary losses of VDBP may portend risk for intrarenal and extrarenal vitamin D deficiencies.

## 1. Introduction

Abnormal excretion of protein is a clinically relevant indicator of impending diabetes-related renal damage; however, mechanisms underlying proteinuria in diabetic nephropathy associated with type 1 diabetes mellitus (T1D) are not completely understood. Numerous studies suggest the involvement of the glomerulus wherein cellular damage and glomerular basement membrane thickening is observed [1]. However, proximal tubule dysfunction also appears to be a central event in promoting protein loss in the diabetic kidney due to enhanced sodium reabsorption and decreased protein reabsorption [1–3]. 

We have recently shown that megalin and cubilin, two multiligand coreceptors expressed in the proximal tubules of the kidney and involved with the reuptake of small-molecular-weight proteins, like albumin, are more abundant in the urine of humans with T1D and microalbuminuria [4]. Many megalin/cubilin ligands, including vitamin-D-binding protein, are also elevated in the urine of these individuals [5]. In support of these findings in humans with T1D, STZ-induced T1D in rats results in decreased megalin protein expression along the apical membrane of the proximal convoluted tubules [6]. Thus, loss of megalin/cubilin could contribute to the inability of proximal tubules to adequately reabsorb many filtered proteins in T1D. 

 Megalin has been shown to play a central role in vitamin D metabolism. Megalin is involved in the uptake of the vitamin-D-binding protein (VDBP)/25-hydroxy vitamin D3 (25-OHD) complex from the glomerular filtrate, where it is endocytosed by the proximal tubule. In the proximal tubule, 25-OHD is dissociated from VDBP and 1-hydroxylated by 1-*α* hydroxylase to form 1,25(OH)_2_D3 (1,25-OHD), which is returned to the circulation [7]. Thus, reduced uptake of VDBP/25-OHD by megalin in diabetic nephropathy could lead to intrarenal losses of VDBP and vitamin D. To better understand the renal response to and the consequences of diminished endocytic ligand functioning in the context of diabetes, we have studied, longitudinally, nephropathy-prone DBA/2J diabetic mice to explore if megalinuria occurs in an albuminuric murine model of T1D and if it is associated with abnormal intrarenal vitamin D homeostasis. 

## 2. Materials and Methods

### 2.1. Modeling of Diabetic Nephropathy in DBA/2J Mice

DBA/2J mice (The Jackson Laboratories, Bar Harbor, ME, USA) were treated with STZ (40 mg/kg/day × 5 days given at 10-11 weeks of age, to induce diabetes) or vehicle (PBS). Mice were provided *ad libitum* access to food and water and monitored weekly for glucosuria and weight change as described elsewhere [8]. Groups of diabetic and control mice were designated for sacrifice at 10, 15, or 18 weeks after the onset of diabetes. Mice were transferred to individual metabolic cages for urine collections 24 to 48 hours before sacrifice. At sacrifice, trunk blood and kidneys from each animal were collected. All procedures and protocols were approved by the Institutional Animal Care and Use Committee at the University of Arkansas for Medical Sciences.

### 2.2. Assays

Urine samples were analyzed for microalbuminuria (Albuwell M ELISA, Exocell, Philadelphia, PA USA) and by Western blot for the presence of megalin (Array Genetics, LLC, Newtown, CT) and VDBP (R&D Systems, Minneapolis, MN USA). Quantitative dot blotting for urine VDBP was carried out as described elsewhere [9]. Urinary 25-OHD was measured in undiluted urine by ELISA (Immunodiagnostics Systems, Fountain Hills, AZ, USA). Serum intact PTH was assayed by ELISA (ALPCO, Salem, NH, USA).

### 2.3. Quantitative Real-Time PCR (qRT-PCR)

RNA was prepared from kidneys, cDNA synthesized, and qRT-PCR performed as described previously [10]. Primer sets for each gene of interest (megalin, cubilin, Txnip, 1-*α* hydroxylase, calbindin D-9k, and Calbindin D-28k) are included in Table 1 in Supplementary material available at doi:10.1155/2011/269378. 

### 2.4. Statistical Analyses

Statistical analyses were performed using GraphPad PRISM 5.02 software. The Mann-Whitney test was used to ascertain differences between groups, and data are presented as mean ± standard error of the mean. Linear relationships between data sets were analyzed using the Pearson Product Moment Correlation. Statistical significance was defined as *P* < .05. 

## 3. Results

Diabetic DBA/2J (STZ-treated) mice assessed at 10, 15, or 18 weeks of diabetes demonstrated severe hyperglycemia, albuminuria (increased albumin/Cr), and weight loss; renal size was significantly increased at 18 weeks of diabetes (Table 1). These data are consistent with the findings of Qi et al. [8] showing that diabetic DBA/2J mice exhibit early and persistent abnormal protein excretion rates and nephromegaly (i.e., nephropathy prone). 

**Figure 1:**
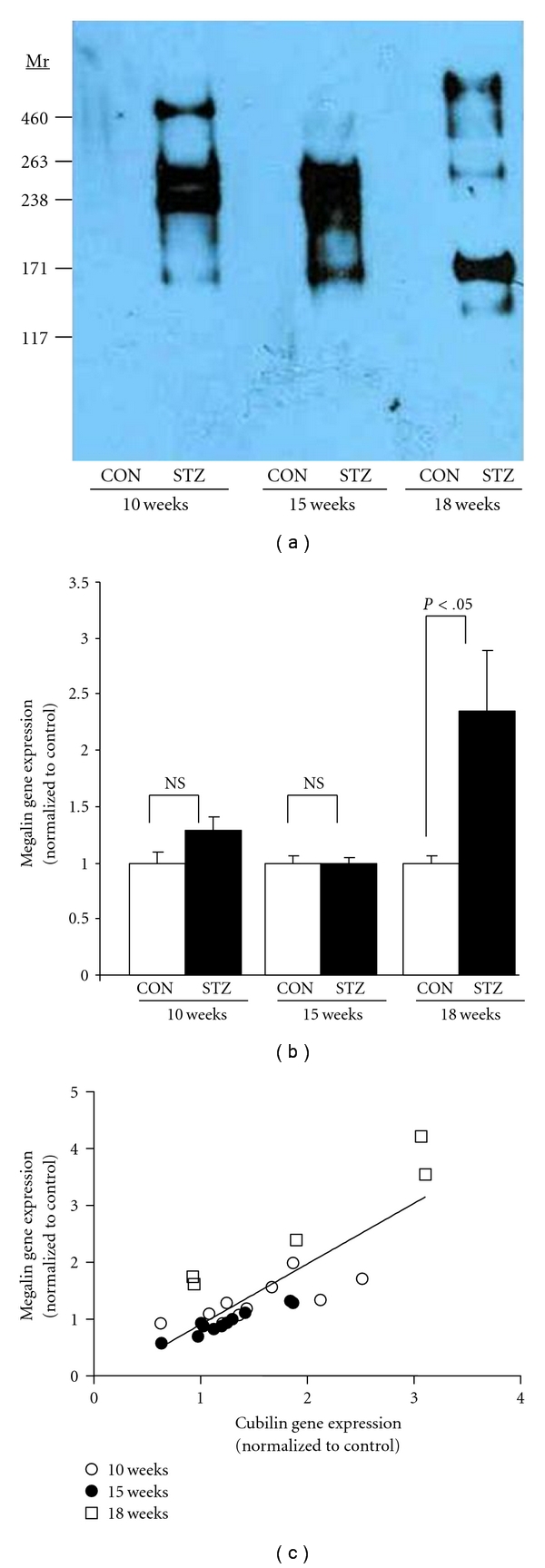
(a) Immunoblotting for the detection of megalin, in the pooled urine of DBA/2J mice without diabetes (CON) or with diabetes (STZ) for 10-, 15-, or 18-week duration (*n* = 6 mice/group, containing equal amounts of creatinine (Cr) for each sample (2.0 *μ*g Cr/lane)). (b) Megalin gene expression as determined by qRT-PCR in renal tissue from control and diabetic DBA/2J mice at 10-, 15-, and 18-week duration. (c) Correlation of megalin and cubilin gene expression in kidneys from diabetic DBA/2J mice (10, 15, and 18 weeks). Immunoblotting and qRT-PCR were carried out as described in Section 2.

Pooled urine from DBA/2J diabetic urine contained immunoreactive megalin at all time points, yet no megalin was detected in the urine from control animals at any time point (Figure 1(a)). Little to no full-length megalin (660 kDa) was detected, suggesting that megalin was excreted primarily as a cleaved product, consistent with megalin fragments observed in human urine [11]. Renal expression of megalin mRNA was also assessed in control and diabetic DBA/2J mice. Figure 1(b) shows that megalin gene expression was similar in control and diabetic kidneys at 10 and 15 weeks after the onset of diabetes. In contrast, after 18 weeks of diabetes, megalin expression increased significantly in the diabetic animals. Megalin and cubilin function as co-receptors involved in the uptake and endocytosis of many small-molecular-weight proteins in the proximal tubule.  Figure 1(c) shows that renal gene expression of the co-receptors megalin and cubilin in DBA/2J kidneys (10–18 weeks) is highly correlated (*r* = 0.8284; *P* < .0001). 

**Figure 2:**
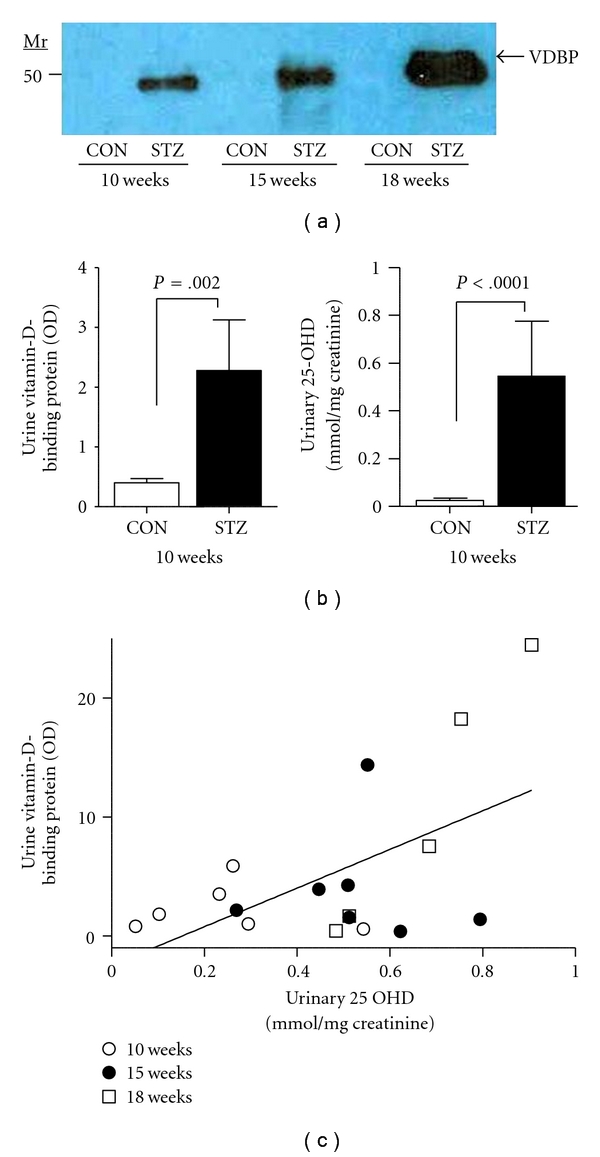
(a) Immunoblotting for the detection of VDBP in the pooled urine of DBA/2J mice without diabetes (CON) or with diabetes (STZ) for 10-, 15-, or 18-week duration (*n* = 6 mice/group, containing equal amounts of creatinine (Cr) for each sample (2.0 *μ*g Cr/lane)). (b) VDBP in urine of control and diabetic mice was assessed by semiquantitative dot blotting, and urine 25-OHD was assayed by ELISA in urine of control and diabetic mice as described in Section 2. (c) Correlation of VDBP and 25-OHD assayed in urine from diabetic DBA/2J mice (10, 15, and 18 weeks—see legend).

Vitamin-D-binding protein (VDBP) uptake in the proximal tubule is principally controlled by megalin [12]. Therefore, if megalin loss of function occurs in the diabetic kidney, then VDBP might be found in greater abundance in the urine of diabetic mice. VDBP was detected at its predicted molecular mass of ~52 KDa in pooled urine of diabetic animals by Western blotting (Figure 2(a)). Little or no VDBP was detected in control urines. VDBP was then assessed by semiquantitative dot blotting in individual urine samples from control and diabetic mice. After 10 weeks of diabetes, diabetic animals excreted elevated amounts of VDBP (Figure 2(b)). Because VDBP excretion in the megalin-null mouse has been associated with urinary losses of 25-OHD, urinary 25-OHD was assessed. Diabetic DBA/2J mice excreted significantly higher amounts of 25-OHD than did control mice after 10 weeks of diabetes (Figure 2(b)). There was a significant association between urinary VDBP, and urinary 25-OHD in diabetic mice (Figure 2(c); *r* = 0.5577; *P* = .0162); however, no significant correlation was found between urinary VDBP and urinary 25-OHD in control mice (*r* = −0.06; *P* = .8). Together, these data show that nephropathy-prone diabetic DBA/2J mice experience early and persistent urinary losses of VDBP and 25-OHD. 

**Figure 3:**
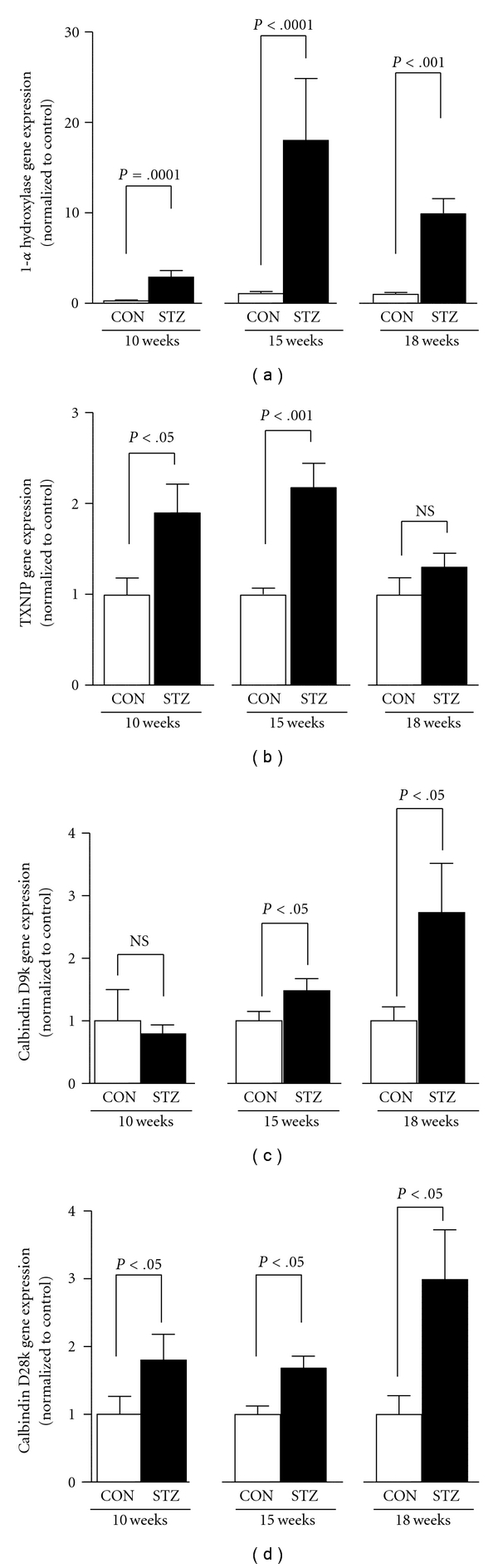
(a) 1-*α* hydroxylase, (b) TXNIP, (c) calbindin D9k, and (d) calbindin D28k gene expression as determined by qRT-PCR in renal tissue from control (CON) and diabetic (STZ) DBA/2J mice at 10-, 15-, and 18-week duration. Quantitative RT-PCR was carried out as described in Section 2.

Because the diabetic DBA/2J mouse excretes significant amounts of megalin, VDBP and 25-OHD, we next examined renal genes involved in vitamin D metabolism. Figure 3(a) shows that 1-*α* hydroxylase, which converts 25-OHD into 1,25-OHD, was upregulated in kidneys of diabetic mice at all three time points examined. Because PTH regulates renal 1-*α* hydroxylase expression, serum PTH was measured in control and diabetic mice at all time points. PTH values were more variable in the diabetic animals, but no statistical difference was found between control and diabetic mice at 10, 15, or 18 weeks after the onset of diabetes (see Supplementary Figure 1). Renal expression of 24-hydroxylase was not altered at any time point in diabetic mice (data not shown). 

Because the upregulation of 1-*α* hydroxylase in the DBA/2J diabetic kidney might portend intracellular accumulation of 1,25-OHD, target genes of 1,25-OHD were also assessed in these mice. Figures 3(b)–3(d) show three target genes known to be regulated by 1,25-OHD in the kidney: thioredoxin-interaction protein (txnip, also known as vitamin D up-regulated protein), calbindin D-9k and calbindin D-28k [13]. All were up-regulated in their expression in the kidneys of diabetic mice. Furthermore, both calbindin species were highly correlated in their levels of expression, suggesting a common pathway of regulation (*r* = 0.7555, *P* < .0001).

## 4. Discussion

Megalin and its coreceptor cubilin function as endocytic receptors for an extensive list of ligands including albumin, vitamin-carrier proteins, lipoproteins, hormones, enzymes, immune-related proteins, and drugs [14]. Thus, alterations in the expression or activity of megalin and/or cubilin may lead to a number of pathological consequences. Megalin-knockout mice exhibit albuminuria as well as urinary losses of low-molecular-weight proteins, including VDBP [15]. In humans, mutations in megalin cause Donnai-Barrow and facio-oculo-acoustico-renal syndrome [16]. These individuals display several phenotypic characteristics similar to megalin knockout animals, including renal disease, proteinuria, and increased urinary VDBP levels. Megalin dysfunction has also been demonstrated in Dent disease, caused by a mutation in the chloride channel *CLCN5*, which results in low-molecular-weight proteinuria and urinary VDBP loss in humans, similar to mice deficient in the *CLCN5 *gene and protein product, CLC-5 [17]. Similarly, we have recently demonstrated increased urinary loses of megalin/cubilin and VDBP in humans with T1D and microalbuminuria [4, 5], suggesting that intrarenal dysregulation of the vitamin D pathway in T1D may mimic that observed in other conditions in which the megalin-mediated endocytosis is disrupted.

We have studied the renal response to T1D in the nephropathy-prone diabetic DBA/2J mouse and have demonstrated that megalin and VDBP are both lost over time in the urine of diabetic animals. Mechanisms involved in the renal loss of megalin and megalin ligands are currently poorly understood; however, previous studies have shown that megalin can be shed from cell surfaces by matrix metalloproteinase activity [18, 19]. Interestingly, MMP activity is elevated in urine from T1D subjects [20] and in the DBA/2J diabetic mouse (data not shown). Thus, enhanced MMP activity in the parenchyma and/or tubular lumen of the diabetic kidney may result in excess shedding and loss of megalin from proximal tubule cell surfaces. As a consequence of megalin deficiency, the megalin ligand, VDBP, and 25-OHD are also lost in the urine of diabetic DBA/2J mice. This is similar to what has been reported for the megalin null mouse and the CLC-5-deficient mouse. Taken together, these data support the concept that impairment of megalin-mediated endocytosis results in the loss of the VDBP/25-OHD complex, irrespective of the underlying cause for megalin dysfunction.

Insulin deficiency, streptozotocin, and/or hyperglycemia could also be potential mediators involved in the loss of megalin ligands, such as VDBP and 25-OHD. To address these variables, we have studied albuminuria and VDBP in the urine from long-term diabetic C57B/6 mice, which do not develop renal pathology, yet develop hyperglycemia, secondary to insulin deficiency after STZ injection, just as the DBA/2J diabetic mouse does [8]. Unlike diabetic DBA/2J mice, no significant albuminuria or enhanced urinary VDBP excretion was observed in diabetic C57B/6 mice compared to control mice (see Supplementary Table 2); yet the diabetic C57B/6 mice were exposed to STZ, experienced loss of beta cell function, and had significant hyperglycemia as was observed in the diabetic DBA/2J mice, suggesting that insulin deficiency, STZ, or hyperglycemia is not directly causal to VDBP urinary losses. 

Renal expression of 1-*α* hydroxylase is significantly elevated in the diabetic DBA/2J mouse; however, several studies have shown in rodent models of STZ-induced diabetes that circulating concentrations of 1,25-OHD are typically decreased [21, 22]. Similarly, the megalin knockout mouse and the CLC-5 knockout mouse demonstrate elevated renal expression of 1-*α* hydroxylase, yet, surprisingly manifest low circulating concentrations of 1,25-OHD [23, 24]. Furthermore, circulating PTH, a primary regulator of renal 1-*α* hydroxylase expression, is not elevated in megalin knock-out mice, CLC-5 knock-out mice, or in DBA/2J diabetic mice. The mechanism behind the paradoxical finding of elevated intrarenal 1-*α* hydroxylase expression in the context of megalin dysfunction has been addressed by Maritzen et al. [24]. In this paradigm, PTH, which is normally retrieved from the urine by megalin, is present at higher concentrations in the lumen of the late proximal tubule, where it activates luminal PTH receptors to increase 1-*α* hydroxylase activity. However, because 25-OHD/VDBP complexes are not efficiently endocytosed by the proximal tubule secondary to megalin dysfunction, lack of substrate leads eventually to diminished 1,25-OHD production. Furthermore, this paradigm predicts that increased concentrations of vitamin D in the lumen of the distal tubule would result in transcriptional upregulation of vitamin D responsive genes. Indeed, several vitamin D responsive genes (i.e., calbindin D-9k, calbindin D-28k, and thioredoxin-interaction protein) were all significantly increased in the kidneys of diabetic mice, similar to what is observed in CLC-5 knock-out mice [24]. Interestingly, calbindin D-28k has been shown to be overexpressed in the distal tubules of diabetic mice [25]. 

Vitamin D is now appreciated as a pleiotropic hormone having affects on multiple tissues and cells [26]. Our findings in diabetic mice and humans [5] of renal wasting of VDBP suggest that abnormal renal metabolism of vitamin D in T1D may have important and broad-spectrum implications. For instance, osteoporosis and fractures are common in T1D [27], as are cardiovascular comorbidities, all of which have been associated with vitamin D deficiency/insufficiency [26]. In addition to the beneficial systemic affects of vitamin D, vitamin D analogs have been shown to prevent renal damage in T1D [28]; however, in the setting of VDBP/25-OHD losses in T1D, vitamin D analog therapy may prove to be less effective. In summary, these studies suggest a mechanism by which intrarenal vitamin D handling and metabolism is altered in diabetic kidney disease, and they signify that urinary losses of VDBP in T1D portend a risk for intrarenal and systemic vitamin D deficiencies. 

## Supplementary Material

The supplementary materials provided show primer sets used for qRT-PCR (Table 1); physical and biochemical characteristics of C57B/6 mice without (control) and with STZ-induced diabetes (diabetes) (Table 2); and PTH concentrations DBA/2J mice without and with STZ-induced diabetes at 10, 15 and 18 weeks off diabetes (Figure S1).

## Figures and Tables

**Table 1:** Characteristics of DBA2/J mice without (control) and with (diabetes) STZ-induced diabetes.

Parameter	10 weeks of DM			15 weeks of DM			18 weeks of DM*		
	DBA/2J Control	DBA/2J Diabetes	*P* value	DBA/2J Control	DBA/2J Diabetes	*P* value	DBA/2J Control	DBA/2J Diabetes	*P* value
*N*	10	10	—	10	10	—	13	6	—
Body weight (g)	28.4 ± 1.3	24.2 ± 0.6	.003	30.6 ± 0.7	23.3 ± 0.8	.0001	28.4 ± 1.2	22.8 ± 1.0	.004
Left Kidney wt (g)	0.27 ± 0.02	0.29 ± 0.01	NS	0.30 ± 0.01	0.30 ± 0.01	NS	0.28 ± 0.01	0.34 ± 0.04	.05
Blood glucose (mg/dl)	104.6 ± 6.9	559.1 ± 9.4	.0001	136.4 ± 9.3	561.9 ± 16.3	.0001	109.9 ± 4.9	582.2 ± 6.7	.0001
Urine albumin/Cr (*μ*g/mg)	52.5 ± 17.4	88.0 ± 21.9	.04	67.8 ± 15.5	178.3 ± 19.7	.001	18.3 ± 3.1	73.6 ± 19.8	.001

*Fewer DBA/2J diabetic mice were available to study at 18 weeks due to increased mortality within this group as described elsewhere [8].
