# Supplementary material for: Dysregulation of the Intrarenal Vitamin D Endocytic Pathway in a Nephropathy-Prone Mouse Model of Type 1 Diabetes
Source: Exp Diabetes Res. 2011 May 16;2011:269378. doi: 10.1155/2011/269378 (PMC3123992; doi:10.1155/2011/269378)
Supplement: Supplementary file 1 — The supplementary materials provided show primer sets used for qRT-PCR (Table 1); physical and biochemical characteristics of C57B/6 mice without (control) and with STZ-induced diabetes (diabetes) (Table 2); and PTH concentrations DBA/2J mice without and with STZ-induced diabetes at 10, 15 and 18 weeks off diabetes (Figure S1). [file 269378.f1.ppt]

## Slide 1
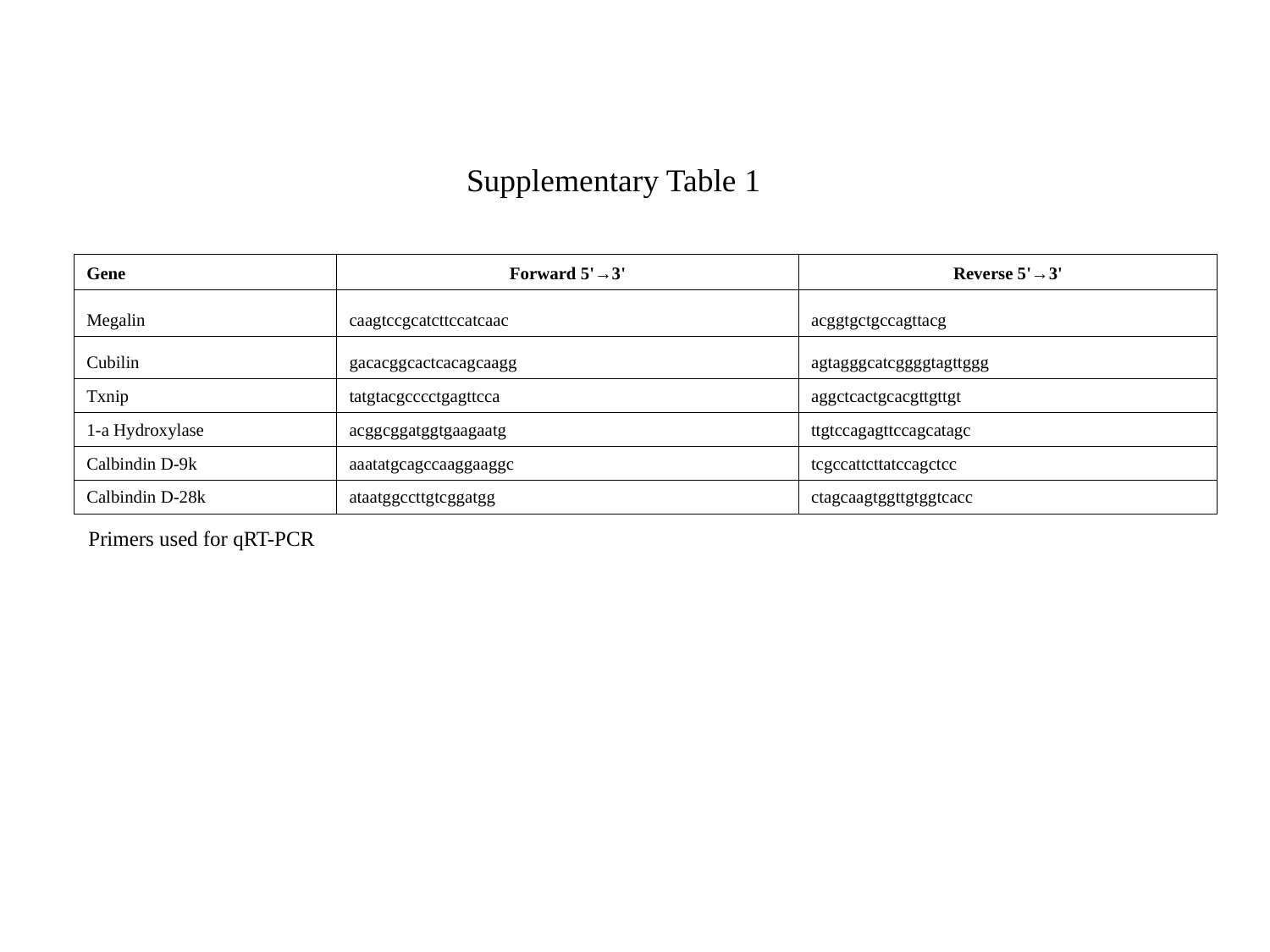

Supplementary Table 1
| Gene | Forward 5'→3' | Reverse 5'→3' |
| --- | --- | --- |
| Megalin | caagtccgcatcttccatcaac | acggtgctgccagttacg |
| Cubilin | gacacggcactcacagcaagg | agtagggcatcggggtagttggg |
| Txnip | tatgtacgcccctgagttcca | aggctcactgcacgttgttgt |
| 1-a Hydroxylase | acggcggatggtgaagaatg | ttgtccagagttccagcatagc |
| Calbindin D-9k | aaatatgcagccaaggaaggc | tcgccattcttatccagctcc |
| Calbindin D-28k | ataatggccttgtcggatgg | ctagcaagtggttgtggtcacc |
Primers used for qRT-PCR

## Slide 2
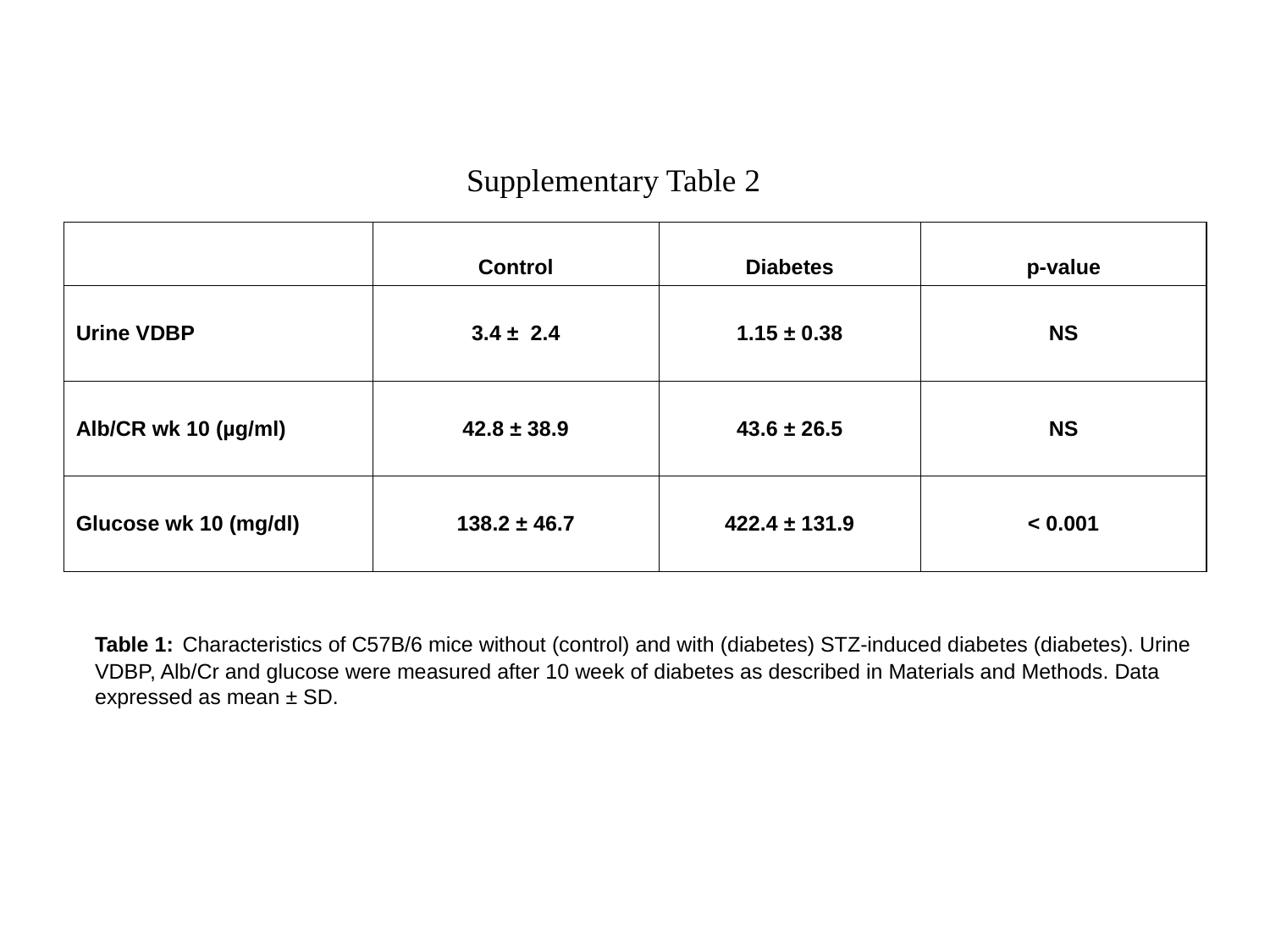

Supplementary Table 2
| | Control | Diabetes | p-value |
| --- | --- | --- | --- |
| Urine VDBP | 3.4 ± 2.4 | 1.15 ± 0.38 | NS |
| Alb/CR wk 10 (µg/ml) | 42.8 ± 38.9 | 43.6 ± 26.5 | NS |
| Glucose wk 10 (mg/dl) | 138.2 ± 46.7 | 422.4 ± 131.9 | < 0.001 |
Table 1: Characteristics of C57B/6 mice without (control) and with (diabetes) STZ-induced diabetes (diabetes). Urine VDBP, Alb/Cr and glucose were measured after 10 week of diabetes as described in Materials and Methods. Data expressed as mean ± SD.

## Slide 3
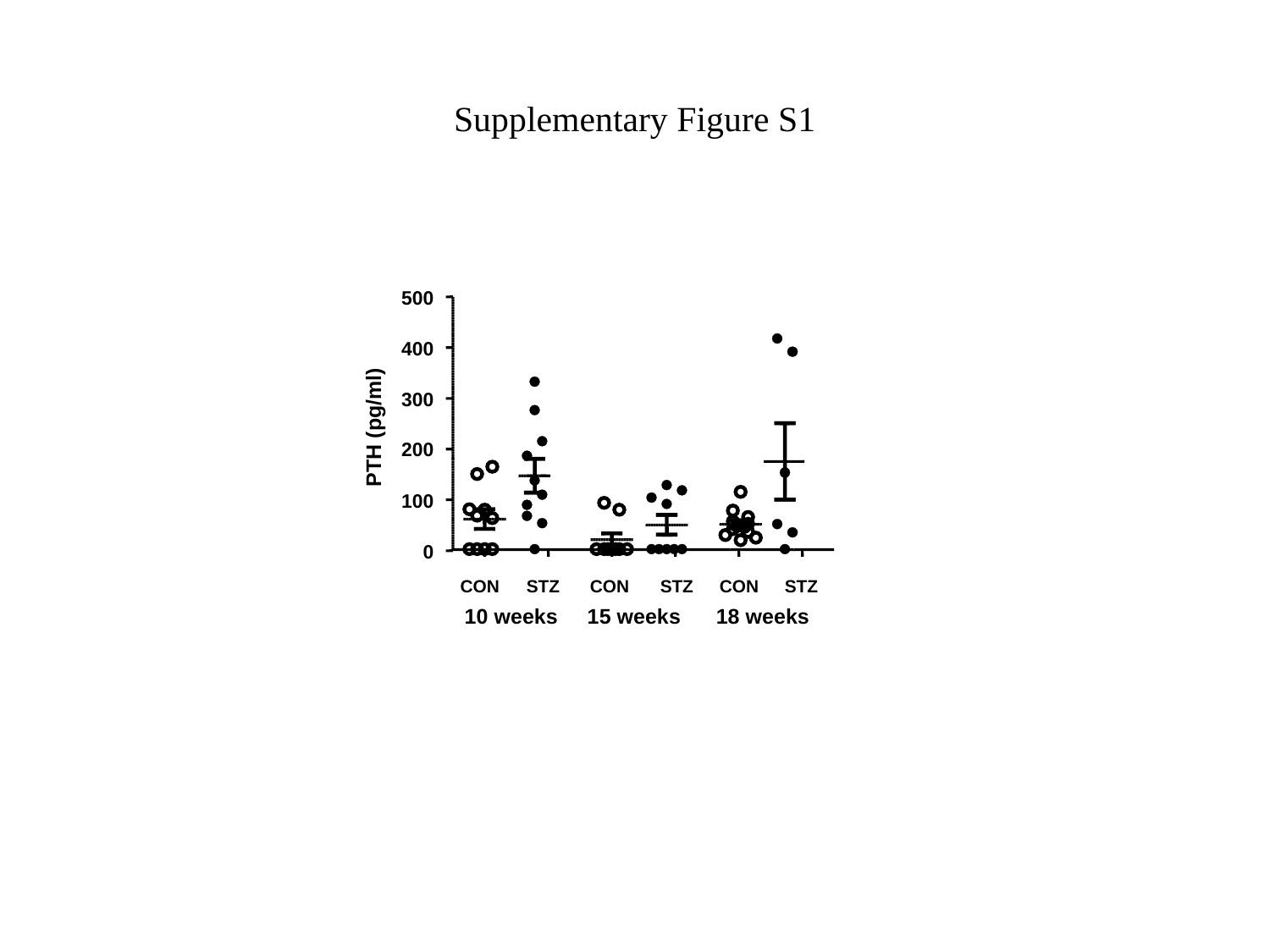

# Supplementary Figure S1
500
400
300
PTH (pg/ml)
200
100
0
CON
STZ
CON
STZ
CON
STZ
 10 weeks 15 weeks 18 weeks
